# Interaction Between the VNTR of the *DAT1* Gene and *DAT1* Methylation in Relation to Impulsivity in Combat Sports Athletes

**DOI:** 10.3390/biomedicines13122893

**Published:** 2025-11-26

**Authors:** Remigiusz Recław, Jolanta Chmielowiec, Krzysztof Chmielowiec, Dariusz Larysz, Agnieszka Pedrycz, Anna Grzywacz

**Affiliations:** 1 Independent Laboratory of Genetics and Behavioral Epigenetics, Pomeranian Medical University in Szczecin, Powstańców Wielkopolskich 72 St., 70-111 Szczecin, Poland; remigiusz.reclaw@pum.edu.pl; 2 Department of Medical Sciences and Public Health, Gdansk University of Physical Education and Sport, Kazimierza Górskiego 1 St., 80-336 Gdansk, Poland; 3 Department of Nursing, Collegium Medicum, University of Zielona Góra, 28 Zyty St., 65-046 Zielona Góra, Poland; j.chmielowiec@inz.uz.zgora.pl; 4 Department of Hygiene and Epidemiology, Collegium Medicum, University of Zielona Góra, 28 Zyty St., 65-046 Zielona Góra, Poland; chmiele@vp.pl; 5 109th Military Hospital with Polyclinic, Ministry of National Defense, ul. Ksiedza Piotra Skargi 9/11, 71-422 Szczecin, Poland; dariuszlarysz@hotmail.com; 6 Faculty of Medicine and Health Sciences, University of Applied Sciences in Tarnow, Mickiewicza 8, 33-100 Tarnów, Poland; apw4@wp.pl

**Keywords:** *DAT1* methylation, *DAT1* VNTR, dopaminergic system, impulsivity, combat sport athletes, epigenetics

## Abstract

**Background**: Dopaminergic signaling is a key mechanism in behavioral regulation and impulse control. While *DAT1* promoter methylation has been linked to behavioral dysregulation in clinical groups, its role in high-functioning populations such as elite athletes remains unclear. **Objectives**: To compare *DAT1* promoter methylation, *DAT1* VNTR genotype, and impulsivity between elite combat sport athletes and matched controls, and to assess potential gene–environment interactions. **Methods**: The study included 209 male participants (100 elite combat athletes, 109 controls). Methylation of 33 CpG sites within the *DAT1* promoter was quantified from peripheral blood DNA. *DAT1* VNTR genotypes were determined via PCR and gel electrophoresis. Impulsivity was assessed using the Barratt Impulsiveness Scale (BIS-11). Group differences and interactions were analyzed using analysis of variance (ANOVA), non-parametric tests, and post hoc comparisons. **Results**: Athletes displayed significantly higher overall *DAT1* promoter methylation and lower impulsivity scores across all BIS-11 subscales compared with controls. A significant group × genotype interaction for methylation indicated genotype-specific epigenetic differences by athletic status. No differences in VNTR genotype or allele frequencies were observed. **Conclusions**: Elevated *DAT1* promoter methylation in elite athletes may be associated with enhanced behavioral control, potentially reflecting neurobiological adaptations to high-intensity training. These results highlight the need to integrate genetic and epigenetic perspectives in sports science. Longitudinal and multi-omics studies are warranted to determine causal links and evaluate the potential of epigenetic markers as indicators of performance-related traits.

## 1. Introduction

Combat sports such as mixed martial arts, boxing, judo, and karate demand not only superior physical conditioning but also refined cognitive and emotional capacities. Elite athletes in these disciplines must operate effectively under extreme pressure, displaying rapid situational assessment, emotional stability, and precise impulse regulation. These characteristics make combat sport practitioners a valuable model for investigating the biological and psychological foundations of self-regulatory abilities in high-functioning individuals exposed to persistent stressors [1,2].

An integrated approach combining genetic, epigenetic, and psychological perspectives provides a multidimensional understanding of individual differences in traits critical to sports performance [3]. While genetic variation can shape neurotransmitter signaling, stress responsiveness, and decision-making dynamics, epigenetic mechanisms such as DNA methylation reflect long-term adaptations to environmental demands. Psychological assessment captures the behavioral output of these processes. Despite this, research simultaneously examining genetic and epigenetic factors in the context of cognitive–emotional traits in elite athletes remains scarce [4].

The dopamine transporter gene (*DAT1*/SLC6A3) contains a 40-base pair variable number tandem repeat (VNTR) polymorphism in its 3′-untranslated region, with the 9-repeat (9R) and 10-repeat (10R) alleles being most common. Functional studies suggest that this VNTR can modulate *DAT1* expression by influencing mRNA regulation, although reported effects vary in both direction and magnitude [5,6]. Neuroimaging studies have also yielded inconsistent associations between VNTR genotype and striatal dopamine transporter availability, implying that the contribution of a single polymorphism may be modest and dependent on environmental or biological context [7]. Meta-analyses in behavioral domains, including ADHD (Attention-Deficit/Hyperactivity Disorder) and impulsivity, support only small effect sizes for this marker [8]. These observations underscore the need to investigate the VNTR within a broader regulatory framework—including epigenetic mechanisms such as promoter methylation—to better understand how genetic and epigenetic variation jointly influence dopaminergic tone in high-performance populations.

*DAT1* promoter methylation can suppress gene expression, thereby modifying dopamine clearance from the synaptic cleft. Aberrant methylation patterns have been associated with psychiatric conditions marked by impaired behavioral control, such as ADHD and substance dependence [9,10]. However, little is known about how promoter methylation contributes to personality and self-regulation traits in healthy individuals functioning at elite competitive levels [11].

In the present study, we examined the combined effects of the *DAT1* VNTR polymorphism and *DAT1* promoter methylation on impulsivity in professional combat sport athletes and matched non-athlete controls. Impulsivity was measured using the Barratt Impulsiveness Scale—Version 11 (BIS-11), which evaluates attentional, motor, and non-planning components [12]. By integrating molecular and psychological data, we sought to clarify the neurobiological mechanisms underlying self-regulation in elite sport contexts.

## 2. Materials and Methods

### 2.1. Participants

This study involved 100 male combat athletes (M = 21.96 years, SD = 4.61) and a control group of 109 age-matched healthy male individuals (M = 21.86 years, SD = 3.85). All participants gave written informed consent after being fully briefed on the study’s aims and procedures. To control for potential confounding due to population structure, only individuals of Caucasian ethnicity from the same region of Poland were included in both groups. Psychiatric screening for both groups was conducted using the Mini International Neuropsychiatric Interview (MINI) to exclude drug addiction, anxiety, depression, and other psychiatric disorders. All participants were instructed to abstain from alcohol and other psychoactive substances for at least 24 h prior to sample collection. Blood samples were collected in the morning hours under standardized laboratory conditions to minimize circadian and environmental variability.

The athlete group consisted of healthy males with no history of psychosis or substance use disorders. Participants were actively involved in one of the following combat sports: mixed martial arts (n = 37), judo (n = 22), boxing (n = 19), karate (n = 14), or kickboxing (n = 8). Recruitment strategies included direct outreach to national-level coaches and athletes, as well as announcements during centralized training camps.

All procedures complied with the ethical principles of the Declaration of Helsinki. Ethical approval was obtained from the Bioethics Committee of the Regional Medical Chamber in Szczecin (Protocol No. 13/KB/VI/2016, dated 8 December 2016).

### 2.2. Measures 

To measure individual differences in impulsivity, the study employed the Barratt Impulsiveness Scale (Version 11, BIS-11) [12]. This 30-item questionnaire requires participants to evaluate how frequently they engage in specific behaviors or thought patterns, using a 4-point scale from “rarely/never” to “almost always/always.” The scale captures three distinct dimensions of impulsivity: attentional (e.g., distractibility and cognitive disorganization), motor (e.g., acting on the spur of the moment), and non-planning (e.g., lack of future orientation or structured thinking). The BIS-11 has been extensively validated and is widely regarded as a gold-standard instrument in research on impulsivity, especially in the context of behavioral regulation and substance-related disorders.

### 2.3. Genotyping and DNA Methylation Assay

Genomic DNA was extracted from peripheral blood collected from combat sport athletes and control participants using a commercial DNA purification kit (A&A Biotechnology, Gdynia, Poland), following the validated procedure described by Recław et al. (2024) [11]. All samples were stored at −20 °C until analysis.

**Figure 1:**
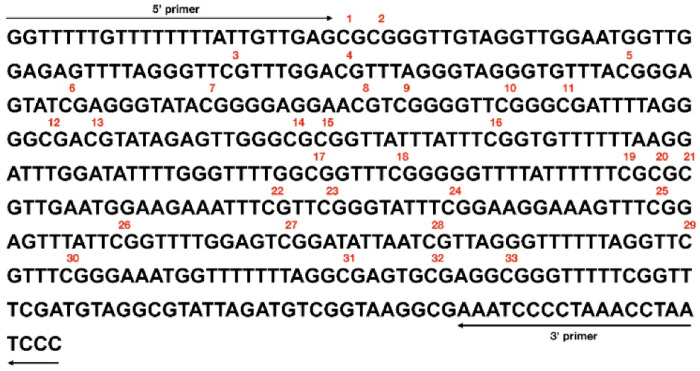
Schematic representation of the analyzed *DAT1* promoter fragment showing primer locations and numbering of CpG sites (red numbers).

For DNA methylation profiling, 250 ng of genomic DNA from each sample was subjected to sodium bisulfite conversion using the EZ DNA Methylation Kit (Zymo Research, Orange, CA, USA), according to the manufacturer’s protocol. Bisulfite-specific PCR was performed on a Mastercycler epgradient S (Eppendorf, Hamburg, Germany) to amplify a 447 bp fragment of the *DAT1* gene promoter region (ENSG00000142319) corresponding approximately to the region between −447 and −1 bp upstream of the transcription start site (TSS; hg38, chr5:1,445,176, minus strand) and encompassing 33 CpG sites within the CpG island upstream of the TSS, with the reverse primer positioned immediately upstream. Primers were designed in MethPrimer (http://www.urogene.org/cgi-bin/methprimer/methprimer.cgi, accessed 29 April 2022) and synthesized by Genomed.pl (Warsaw, Poland): forward DATF 5′-GGTTTTTGTTTTTTTTATTGTTGAG-3′ and reverse DATR 5′-AAATCCCCTAAACCTAATCCC-3. Because primer design was optimized for bisulfite conversion efficiency (minimizing cytosine content outside CpG sites) rather than for precise transcript coverage, the TSS position should be considered approximate and is indicated in the text only for genomic reference. Figure 1 presents the bisulfite-converted sequence, CpG site positions (1–33), and primers. 

PCR reactions were prepared in 25 µL volumes containing 100 ng of bisulfite-converted DNA, 10 pmol of each primer, 50 mM KCl, 10 mM Tris-HCl (pH 8.3), 1.5 mM MgCl_2,_ 200 µM each dNTP, and 0.8 U of Taq DNA polymerase. The cycling protocol consisted of an initial denaturation at 94 °C for 5 min, followed by 30 cycles of denaturation at 94 °C for 55 s, annealing at 55 °C for 50 s, and extension at 72 °C for 1 min, with a final extension at 72 °C for 10 min.

The resulting amplicons were sequenced using the BigDye Terminator v3.1 Cycle Sequencing Kit (Applied Biosystems, Foster City, CA, USA) on an ABI Prism 3130XL Genetic Analyzer (Applied Biosystems, Foster City, CA, USA). Chromatograms were inspected in 4Peaks software, version 1.8 (Nucleobytes, Aalsmeer, The Netherlands). The methylation status of each CpG site was determined from bisulfite sequencing chromatograms as the ratio of cytosine (G) to thymine (A) peaks, calculated as G/(G + A). A CpG site was classified as methylated when the G/(G + A) peak height ratio exceeded 20%, a threshold applied solely to exclude background noise in Sanger sequencing chromatograms and not to indicate full methylation in the heterogeneous blood cell population. Methylation levels below this cut-off are generally regarded as technical signal variation rather than true methylation, and this criterion has been widely validated in bisulfite-based assays performed in molecular biology laboratories operating under EN ISO 15189 standards. Each site was therefore coded as methylated (1) or unmethylated (0).

For each participant, two complementary indices were computed: (i) the percentage of methylated CpG sites, calculated as the proportion of loci exceeding the 20% threshold ((number of methylated sites ÷ 33) × 100), and (ii) the total number of methylated CpG sites (range: 0–33). Both indices were used in subsequent statistical analyses to provide a binary-based, reproducible representation of *DAT1* promoter methylation. Each CpG site was numbered sequentially (CpG1–CpG33) based on its position relative to the transcription start site, and detailed methylation levels for each CpG site are provided in Table S1.

*DAT1* VNTR genotyping in the 3′-untranslated region was performed by conventional PCR using the following primers: forward 5′-TGT GGT GTA GGG AAC GGC CTG AG-3′ and reverse 5′-CTT CCT GGA GGT CAC GGC TCA AGG-3′. Reaction mixtures were prepared as above, and amplification products were separated by electrophoresis on 3% agarose gels containing ethidium bromide. Gels were run on a horizontal gel electrophoresis unit (Bio-Rad, Hercules, CA, USA) at 90 V in 1× TAE buffer for 90 min. Bands were visualized under ultraviolet illumination and compared with a DNA ladder (100 bp, Thermo Fisher Scientific, Waltham, MA, USA). The expected product sizes were 450 bp for the 10-repeat allele and 410 bp for the 9-repeat allele [13].

### 2.4. Statistical Analysis

Hardy–Weinberg equilibrium (HWE) for *DAT1* VNTR genotype distributions was tested separately in athletes and controls using an online calculator (https://wpcalc.com/en/equilibrium-hardy-weinberg/, accessed 12 December 2024). Differences in genotype and allele frequencies between groups were examined using the chi-square test. Between-group differences in BIS-11 scores and the number of methylated CpG sites were assessed with the Mann–Whitney U test due to non-normal variable distributions. A two-way ANOVA was applied to evaluate the effects of group, genotype, and their interaction on BIS-11 subscales and *DAT1* methylation, with Levene’s test confirming variance homogeneity (*p* > 0.05). Post hoc analyses were performed using Fisher’s LSD test. Results from a robust two-way ANOVA on aligned rank-transformed data yielded the same pattern of significance, confirming that the findings were not dependent on the normality assumption. Effect sizes are reported as partial eta squared (η^2^). 

For the variables related to BIS-11 Subscale Scores and *DAT1* Gene Methylation Site Counts, a significance level of 0.01 (0.05/5) was adopted, using the Bonferroni correction for multiple comparisons. For the variables related to methylation of the 33 CpG sites included in the Supplementary Materials, a significance level of 0.0015 (0.05/33) was adopted, using the Bonferroni correction for multiple comparisons.

DNA methylation data were obtained from an accredited laboratory operating in accordance with EN ISO 15189 standards, which establish international requirements for quality management systems, validation of analytical methods, and control of measurement accuracy and reproducibility in medical laboratories (International Organization for Standardization. EN ISO 15189:2022—Medical laboratories—Requirements for quality and competence. Geneva, Switzerland: ISO; 2022). Data were directly provided in a binary format (methylated = 1, unmethylated = 0), based on a 20% methylation threshold per CpG site, which served as a technical noise filter. Two indices were used in the analyses: (i) the total number of methylated CpG sites (range: 0–33) and (ii) the percentage of methylated CpG sites, reflecting the proportion of loci exceeding this threshold. Analyses were conducted in STATISTICA 13 (Tibco Software Inc., Palo Alto, CA, USA) and JASP version 0.95.2 (JASP Team, University of Amsterdam, Amsterdam, The Netherlands) for Windows 11 Pro.

## 3. Results

### 3.1. DAT1 VNTR Distribution and Hardy–Weinberg Equilibrium

Genotype distributions in both the combat sport athlete and control groups conformed to Hardy–Weinberg equilibrium (HWE; *p* > 0.05 in both cases; see Table 1).

### 3.2. Group Differences in DAT1 Promoter Methylation

Taken together, these results indicate that the two groups do not differ in the *DAT1* VNTR structure (genotypes or alleles), ruling out VNTR imbalance as a potential confounder for subsequent analyses. We therefore next examined between-group differences in *DAT1* promoter methylation and their associations with impulsivity measures.

No significant differences were observed in the distribution of *DAT* genotypes between combat sport athletes and controls (10R/10R: 0.46 vs. 0.56; 9R/10R: 0.45 vs. 0.37; 9R/9R: 0.09 vs. 0.07; χ^2^ = 2.0720, *p* = 0.3549). Similarly, the frequencies of individual *DAT* alleles did not differ significantly between the two groups (allele 10: 0.68 vs. 0.74; allele 9: 0.32 vs. 0.26; χ^2^ = 1.7301, *p* = 0.1884; Table 2).

**Figure 2:**
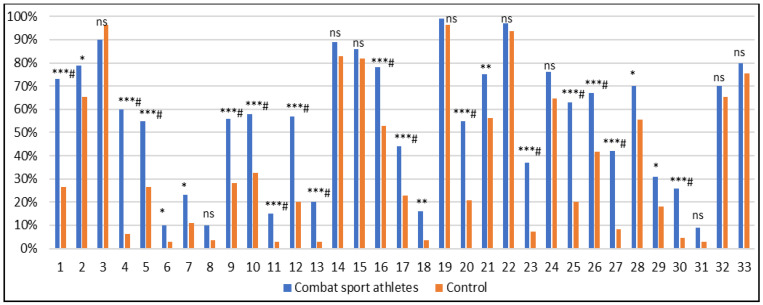
Percentage comparison of methylation levels at individual CpG sites within the *DAT1* gene between combat sport athletes and control participants; ns—not significant (*p* > 0.05); *—*p* ≤ 0.05; **—*p* ≤ 0.01; ***—*p* ≤ 0.001. # Bonferroni correction was applied, and the significance threshold was adjusted to *p* = 0.0015 (0.05/33, number of statistical tests performed).

Table 3 presents the means and standard deviations for all BIS subscales, as well as the number of methylation sites in the *DAT1* gene, for both the combat sport athlete and control groups. Detailed methylation values for each CpG site are provided in Supplementary Table S1. After Bonferroni correction for 33 tests (α = 0.0015), higher methylation was observed at CpG sites 1, 2, 4–7, 9–13, 16–18, 20, 21, 23, and 25–30 in athletes vs. controls (Figure 2, Supplementary Table S1).

Taken together, these results indicate a distinct methylation profile in combat sport athletes, characterized by consistently elevated methylation across multiple CpG loci within the *DAT1* promoter. This suggests that epigenetic modulation of *DAT1* may be more pronounced in athletes, potentially reflecting adaptive responses to long-term physical or psychological stress exposure. These differences were specific to methylation profiles, as no between-group differences in *DAT1* VNTR distribution were observed (see Section 3.1).

### 3.3. Relationship Between Methylation and Lmpulsivity

Combat sport athletes showed a significantly higher number of methylation sites in the *DAT1* gene compared with controls (17.42 vs. 11.74; Z = 7.022; *p* < 0.0001). In contrast, they scored lower on all BIS-11 domains—attentional, motor, and non-planning impulsivity—as well as on the total score (*p* = 0.0136; Table 3). These findings indicate an inverse association between impulsivity and *DAT1* methylation levels, suggesting that higher promoter methylation may be linked to reduced impulsivity in athletes.

**Figure 3:**
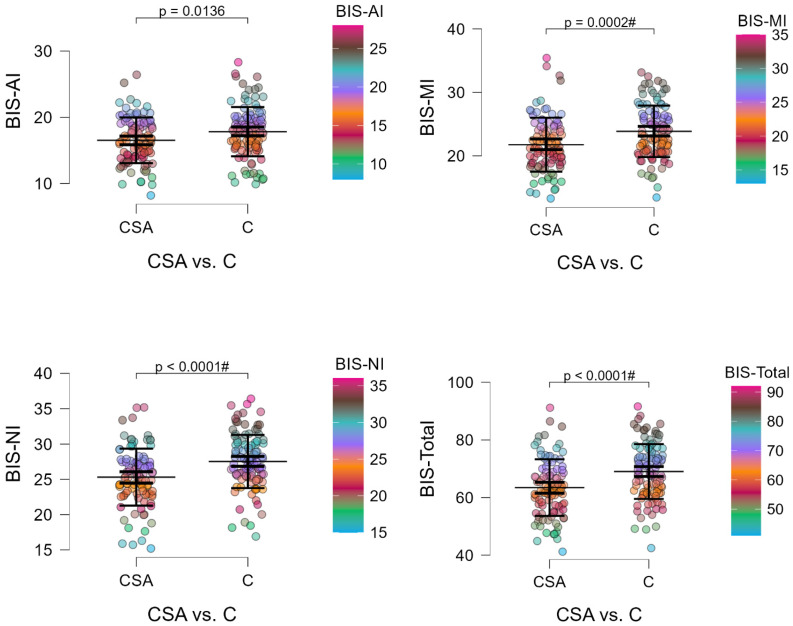
Raincloud plots illustrating BIS-11 subscale scores (Attentional, Motor, Non-planning, and Total) in combat sport athletes (CSA) and control participants (C). Each point represents an individual data value. The long horizontal line indicates the mean, the thick vertical bar shows the 95% confidence interval (CI), and the thin vertical bar represents the standard deviation (SD). # Bonferroni correction was applied, and the *p*-value was lowered to 0.01 (*p* = 0.05/5 number of statistical tests performed).

**Figure 4:**
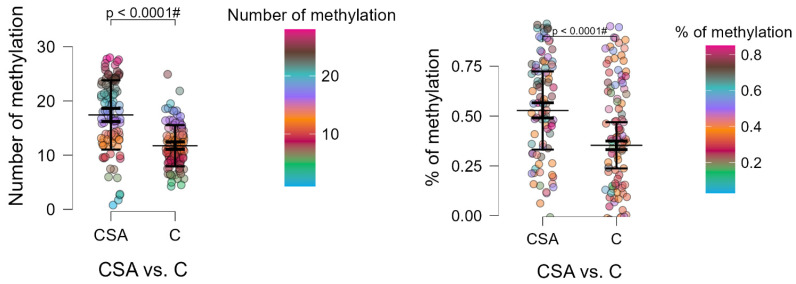
Raincloud plots showing the number and percentage of methylated CpG sites within the *DAT1* promoter in combat sport athletes (CSA) and control participants (C). Each point corresponds to an individual participant’s value. The long horizontal line indicates the mean, the thick vertical bar shows the 95% confidence interval (CI), and the thin vertical bar represents the standard deviation (SD). # Bonferroni correction was applied, and the *p*-value was lowered to 0.01 (*p* = 0.05/5 number of statistical tests performed).

To further illustrate these associations, Figure 3 and Figure 4 visualize the distribution of impulsivity (BIS-11 subscales) and *DAT1* promoter methylation across individual participants. These raincloud plots highlight group differences while preserving information about individual data variability, confirming lower impulsivity and higher methylation levels among combat sport athletes.

Figure 3 highlights the behavioral pattern observed in combat sport athletes, who demonstrated lower impulsivity scores across all BIS-11 dimensions. To explore whether these behavioral traits correspond with molecular differences, Figure 4 presents the distribution of *DAT1* promoter methylation, showing elevated methylation levels in athletes compared with controls. This pattern suggests that epigenetic regulation may underlie these behavioral differences, prompting further analyses of *DAT1* methylation and its interaction with genotype.

The outcomes of the 2 × 3 factorial ANOVA for the Barratt Impulsiveness Scale (BIS) and the number of methylation sites in the *DAT1* gene are presented in Table 4.

For the number of methylation sites, a significant difference was observed between combat sport athletes and controls (F_1,204_ = 17.31; *p* < 0.0001; η^2^ = 0.078). Significant variation was also found across *DAT1* VNTR genotypes (F_2,204_ = 6.05; *p* = 0.0028; η^2^ = 0.056).

**Figure 5:**
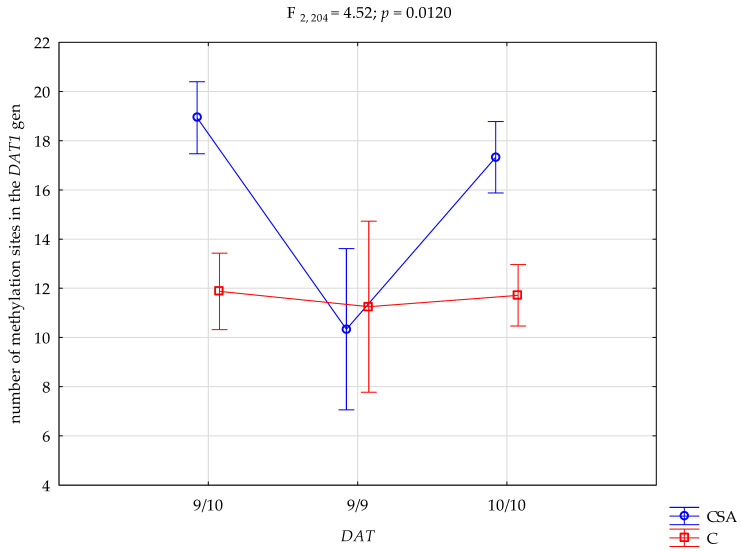
Interaction Between *DAT1* VNTR Genotype and Study Group on the Number of Methylation Sites in the *DAT1* Gene. CSA—combat sport athletes; C—control group.

Moreover, there was a significant interaction between group (athletes vs. controls) and genotype on the number of methylation sites (F_2,204_ = 4.52; *p* = 0.0120; η^2^ = 0.042; Figure 5). The observed power for this interaction was 76%, with the genotype–group interaction explaining approximately 4% of the variance in methylation site counts.

Post hoc comparisons (Table 5) showed that athletes with the 10R/10R genotype had significantly more methylation sites than athletes with the 9R/9R genotype, as well as more than controls with any genotype (10R/10R, 9R/10R, or 9R/9R). Athletes carrying the 9R/10R genotype also had significantly more methylation sites than athletes with the 9R/9R genotype and all control subgroups.

Taken together, these results demonstrate genotype-specific patterns of *DAT1* promoter methylation and their association with impulsivity traits in combat sport athletes.

## 4. Discussion

Although the 20% threshold reliably distinguished signal from noise in Sanger sequencing, average methylation levels across individual CpG sites remained low to moderate (<40%), consistent with partial methylation within a heterogeneous blood cell population. Combat sport athletes showed a significantly higher mean number of methylated CpG sites within the *DAT1* promoter compared to controls (17.42 ± 6.40 vs. 11.74 ± 3.80; *p* < 0.0001), representing a non-trivial main group effect (η^2^ = 0.078). A significant group × genotype interaction (η^2^ = 0.042) indicated that methylation patterns differed according to both athletic status and *DAT1* VNTR genotype, with the highest levels in athletes carrying the 9R/10R genotype, followed by 10R/10R, and the lowest in 9R/9R carriers. These findings point to genotype-dependent differences in *DAT1* methylation associated with athletic status. However, not all of the 33 examined CpG sites exhibited uniform group differences, which likely reflects site-specific variability in regulatory sensitivity within the *DAT1* promoter. This underscores the importance of locus-level analyses in epigenetic studies, as aggregate methylation indices may mask biologically meaningful regional patterns. As shown in Supplementary Table S1, these site-specific variations contributed to the overall between-group difference in methylation levels. Prior work shows that long-term physical training can influence DNA methylation in humans [14,15,16,17].

Athletes scored significantly lower than controls on all BIS-11 sub-scales, with no group × genotype interactions, indicating that reduced impulsivity is likely independent of the *DAT1* VNTR genotype. Consistent with this, the main effect of genotype on BIS-11 scores was non-significant across models. This reduction, together with higher promoter methylation in athletes, is consistent with prior reports linking structured athletic training to changes in dopaminergic regulation [14,15,16,17], although the present findings should be interpreted as correlational.

The lack of genotype frequency differences between groups underscores the importance of considering combined genetic and epigenetic data rather than genetic variation alone. This aligns with evidence that gene–environment processes shape behavioral traits and that DNA methylation has been suggested to mediate links between environmental demands and performance-related cognitive attributes [15,17].

Previous research on the *DAT1* VNTR polymorphism and promoter methylation has primarily targeted clinical populations—especially ADHD and substance use disorders. The present findings extend this line of research by documenting systematic between-group differences in a non-clinical, athletic cohort. In particular, athletes carrying the 9R/10R genotype showed the highest methylation, followed by 10R/10R, with the lowest levels in 9R/9R carriers, consistent with reports that DNA methylation is sensitive to sustained environmental demands, including prolonged training [14,15,16]. While our cross-sectional design precludes causal inference, observing this pattern in an elite, non-clinical context suggests a potential extension of earlier work and aligns with broader work indicating that exercise can modify DNA methylation in humans [14,16].

The lack of group differences in *DAT1* VNTR genotype frequencies aligns with evidence that the main effects of this polymorphism on behavioral phenotypes are small [8] (and with imaging work indicating modest effects on striatal *DAT* availability [6]). In contrast, the significant group × genotype interaction for promoter methylation suggests that *DAT1* variation may gain functional relevance when considered alongside epigenetic regulation [15]. Whether these differences reflect training history, pre-existing predispositions, or residual confounding remains unresolved. In particular, because methylation analysis was performed by an external laboratory using Sanger bisulfite sequencing and we received only binary (methylated/unmethylated) results for each CpG site, we were unable to perform cell-type deconvolution or adjust for blood cell-type heterogeneity—a limitation increasingly recognized in peripheral-blood epigenetic studies.

Regarding impulsivity, athletes showed consistently lower BIS-11 scores across attentional, motor, and non-planning domains relative to controls, in line with prior reports linking structured athletic training to improved self-regulatory and attentional control [1,2]. Although few studies have evaluated *DAT1* methylation and impulsivity concurrently [9,11], the co-occurrence in our sample of higher promoter methylation and lower impulsivity is compatible with a shared neurobiological pathway involving dopaminergic regulation. Promoter methylation can down-regulate *DAT1* expression—as reported in prior studies [18,19,20]—potentially prolonging synaptic dopamine signaling and supporting cognitive focus and inhibitory control under high demands. Given the study design, these associations should be interpreted as correlational; longitudinal and multi-omics approaches are needed to determine temporal ordering and mechanism.

Mechanistically, increased *DAT1* promoter methylation—linked in prior studies to altered dopamine transporter availability [9,10]—may modulate synaptic dopamine signaling and thereby support focus, rapid decision-making, and adaptive motor control in elite combat sports. In our data, methylation was highest in 9R/10R carriers, intermediate in 10R/10R, and lowest in 9R/9R, a genotype-specific pattern consistent with a gene–epigenome interaction model in which *DAT1* VNTR variation influences susceptibility to environmentally induced methylation changes [20]. Prior work, including in vitro studies on DAT1 VNTR–dependent regulation of transporter density [5], molecular analyses in clinical populations [11], and neuroimaging-based assessments of dopaminergic function [21], suggests that individuals with the 10R/10R genotype may exhibit higher baseline transporter expression. In such cases, methylation-based modulation could be advantageous for balancing arousal and inhibitory control, while training-related environmental demands may further shape methylation profiles at dopamine-related loci [14,15,16,17]. It should be emphasized, however, that these mechanistic interpretations are speculative and not directly tested in the present study. As this is a cross-sectional study, causality cannot be inferred, and alternative explanations—including gene–environment correlation or unmeasured confounders—cannot be ruled out.

From a behavioral genetics perspective, the co-occurrence in our sample of higher *DAT1* promoter methylation and lower impulsivity is compatible with the broader view that dopaminergic regulation contributes not only to reward sensitivity and motivation but also to self-regulatory traits essential for elite performance [1,2,22]. This profile—strong approach motivation combined with effective behavioral inhibition—has been described in high-level athletes [1,2,17], and our results provide molecular data that are consistent with, but do not directly prove, this characterization. Nevertheless, further work integrating epigenetic, transcriptomic, and neuroimaging approaches will be necessary to clarify how variation at the molecular level translates into functional brain activity and, ultimately, into behavioral performance in the sporting context [23].

These findings carry practical implications for both sports science and behavioral genetics. In applied sports settings, the observed association between increased *DAT1* promoter methylation and lower impulsivity suggests, but does not demonstrate, that targeted training protocols, psychological interventions, or lifestyle factors influencing epigenetic regulation could contribute to optimizing cognitive control in athletes [14,15,17]. While the present results do not establish causality, they highlight the potential for epigenetic markers to serve as candidate indicators of long-term adaptation to high-performance environments—an idea that would require validation in longitudinal studies examining the stability of methylation patterns over time [11,15,24]. Future research should build on these findings by integrating longitudinal sampling, diverse participant cohorts, and multi-omics approaches combining DNA methylation, transcriptomics, and neuroimaging. Such designs would allow for more precise mapping of the causal pathways linking genetic variation, epigenetic regulation, and behavioral performance in elite athletes. In addition, experimental studies—such as controlled manipulations of training load, psychological stress, or recovery protocols—could help determine whether specific aspects of the athlete environment actively shape the methylation landscape of dopamine-related genes [25]. These studies should also incorporate rigorous control of potential confounding variables (e.g., diet, circadian timing, substance use, blood cell-type composition) and, where possible, assess methylation patterns in both peripheral tissues and relevant target tissues (e.g., skeletal muscle) to better link molecular variation with performance-related physiology.

Future work should directly assess whether VNTR status and promoter methylation relate to *DAT1* transcription. Given the tissue specificity of *DAT1*, this will likely require neural models (e.g., induced pluripotent stem cell–derived dopaminergic neurons) or neuron-enriched biomarkers (e.g., neuron-derived extracellular vesicles from plasma) rather than bulk peripheral blood. As a pragmatic interim step, peripheral pathway readouts (e.g., expression of dopamine-related genes such as *DRD2*, *COMT*, and *MAOA* in sorted leukocyte subsets) could be explored to provide convergent evidence. While highly preliminary, these findings raise the possibility that epigenetic markers could eventually be developed as indicators of long-term adaptation to training load and competitive pressure. However, these results do not establish a causal relationship. Longitudinal and multi-omics studies are needed to verify whether, and to what extent, the training environment shapes *DAT1* methylation and whether such changes translate into lasting differences in impulse control and athletic performance.

Several limitations should be acknowledged. First, the cross-sectional design precludes the determination of whether elevated methylation is a consequence of long-term training or a pre-existing characteristic that predisposes individuals to excel in combat sports. Longitudinal designs tracking athletes over their training careers, ideally beginning before elite-level specialization, would help disentangle these possibilities [11,24,25]. Second, the study included only young adult males of Caucasian ethnicity from a single geographic region; while this choice reduced variability linked to sex-related hormonal fluctuations, it substantially limits the generalizability of our findings. Epidemiological studies consistently report higher prevalence rates of behavioral addictions among males [26,27], which partly explains the recruitment feasibility and rationale for focusing on a homogeneous male cohort. Nevertheless, sex differences in impulsivity, dopaminergic regulation, and epigenetic profiles are well documented, and the exclusion of female participants should be considered a major limitation. Future studies should replicate the present findings in female cohorts and explore potential sex-specific mechanisms underlying the interaction between *DAT1* methylation, genotype, and impulsivity. Third, the study did not assess *DAT1* expression at the mRNA or protein level, nor did it incorporate neuroimaging or neurotransmitter measurements, meaning that functional inferences about the consequences of methylation remain indirect [9,10]. Another limitation is that methylation levels were assessed using bisulfite sequencing, which is considered a semi-quantitative method. While this approach enabled us to examine a relatively broad promoter fragment encompassing 33 CpG sites, the absence of validation using a fully quantitative technique such as bisulfite pyrosequencing represents a limitation. Future studies should incorporate such methods to confirm and refine the present findings. In addition, DNA methylation is tissue-specific. Because *DAT1* is predominantly expressed in the central nervous system, methylation patterns measured in peripheral blood may not fully reflect neural regulation. Nevertheless, peripheral blood is a widely used and practical surrogate tissue in behavioral genetics and sports science. In addition, the 20% methylation threshold applied in this study should be interpreted as a technical noise filter inherent to bisulfite sequencing, not as a biological indicator of full methylation. This methodological approach is widely accepted in human epigenetic and neurobehavioral research, as direct analysis of methylation in brain tissue is ethically and practically unfeasible. Numerous independent studies investigating dopaminergic gene regulation, including *DAT1*, have successfully employed peripheral blood as a valid and reproducible biomaterial for assessing methylation signatures associated with behavioral phenotypes [9,10,17,20]. Moreover, evaluating *DAT1* expression was beyond the scope of the present design because *DAT1* is predominantly expressed in neural tissue and is only weakly detectable in peripheral blood, which was our available biospecimen. While evaluating *DAT1* expression was beyond the scope of this case–control design, future studies integrating methylation and gene expression analyses will be essential to clarify the functional mechanisms underlying the observed epigenetic differences. Finally, potential confounding variables known to influence peripheral DNA methylation—such as smoking status, caffeine or alcohol consumption, circadian timing of sampling, and blood cell-type composition—were not assessed and should be taken into account in future research. Most importantly, the use of outsourced Sanger-based bisulfite sequencing provided only binary methylation calls and precluded the application of reference-based or reference-free cell-type deconvolution algorithms that are currently considered best practice for blood-derived DNA-methylation data. Although this methodological approach has been successfully used numerous times in the dopaminergic epigenetic literature (including our previous high-impact publications), we fully acknowledge that the lack of raw sequencing traces and inability to adjust for leukocyte composition represent a limitation of the present study. Future work from our laboratory will employ in-house next-generation bisulfite sequencing (or pyrosequencing) to enable full statistical adjustment for cell-type proportions and other technical covariates. Despite these limitations, the present study provides novel evidence on epigenetic modulation of the dopaminergic system in elite athletes.

## 5. Conclusions

In this study, combat sport athletes exhibited a significantly higher number of methylated CpG sites in the *DAT1* promoter compared to the control group, alongside lower impulsivity scores across all BIS-11 subscales. The effect on methylation was modulated by the VNTR genotype (significant group × genotype interaction), whereas no interaction was observed for BIS-11 scores. Taken together, these findings suggest that in high-performance athletes, epigenetic regulation of the dopamine transporter may be associated with enhanced behavioral control.

No differences were found in *DAT1* VNTR genotype and allele frequencies between groups, emphasizing the importance of integrating genetic variants with epigenetic modifications rather than analyzing them in isolation. 

## Figures and Tables

**Table 1:** Distribution of *DAT1* VNTR Genotypes and Hardy–Weinberg Equilibrium in Combat Sport Athletes and Control Participants.

	*DAT1* VNTR	Observed (Expected)	allele freq	χ^2^ (*p*-Value)
Combat sport athletes n = 100	10R/10R	46 (46.9)	*p* (10) = 0.68 *q* (9) = 0.32	0.183 (0.6690)
	9R/10R	45 (43.2)		
	9R/9R	9 (9.9)		
Control n = 109	10R/10R	61 (60.2)	*p* (10) = 0.74 *q* (9) = 0.26	0.1641 (0.6854)
	9R/10R	40 (41.6)		
	9R/9R	8 (7.2)		

*p*-value—statistical significance χ^2^ test; *p* and *q* represent the allele frequencies in Hardy–Weinberg equilibrium.

**Table 2:** Prevalence of *DAT1* VNTR Genotypes and Allele Frequencies in Combat Sport Athletes and Control Participants.

*DAT* VNTR					
	Genotypes			Alleles	
	10R/10R n (%)	9R/10R n (%)	9R/9R n (%)	10 n (%)	9 n (%)
Combat sport athletes n = 100	46 (46.00%)	45 (45.00%)	9 (9.00%)	137 (68.50%)	63 (31.50%)
Control n =109	61 (55.96%)	40 (36.70%)	8 (7.34%)	162 (74.31%)	56 (25.69%)
χ^2^ (*p*-value)	2.0720 (0.3549)			1.7301 (0.1884)	

n—number of subjects; *p*-value—statistical significance χ^2^ test.

**Table 3:** BIS-11 Subscale Scores and *DAT1* Gene Methylation Site Counts in Combat Sport Athletes and Control Participants.

BIS-11 Number of Methylation Sites in the *DAT1* Gene	Combat Sport Athletes n = 100	Control n = 109	Z	(*p*-Value)
BIS-AI	16.53 ± 3.45	17.83 ± 3.72	−2.468	0.0136 *
BIS-MI	21.76 ± 4.26	23.86 ± 4.06	−3.763	0.0002 *#
BIS-NI	25.31 ± 4.03	27.53 ± 3.77	−4.131	<0.0001 *#
BIS-Total	63.46 ± 9.83	69.05 ± 9.53	−4.008	<0.0001 *#
Number of methylation sites in the *DAT1* gene	17.42 ± 6.40	11.74 ± 3.80	7.022	<0.0001 *#

*p*—statistical significance with Mann–Whitney U-test; n—number of subjects; M ± SD—mean ± standard deviation; *—statistically significant differences. # Bonferroni correction was applied, and the *p*-value was lowered to 0.01 (*p* = 0.05/5 number of statistical tests performed).

**Table 4:** Two-Way ANOVA Results for BIS-11 Scores and *DAT1* Methylation Across Genotypes and Study Groups.

BIS-11	Group	*DAT1*				ANOVA		
		10R/10R n = 107 M ± SD	9R/10R n = 85 M ± SD	9R/9R n = 17 M ± SD	Factor	F (*p* Value)	η^2^	Power (α = 0.05)
BIS-AI	CSA; n = 100	16.16 ± 3.51	16.93 ± 3.45	16.33 ± 3.35	intercept	F_1,204_ = 2523.28 (*p* < 0.0001) *	0.926	1.000
	Control; n = 109	17.52 ± 4.01	18.30 ± 3.34	17.75 ± 3.41	CSA/control	F_1,204_ = 4.10 (*p* = 0.0442) *	0.020	0.522
					*DAT*	F_2,204_ = 1.09 (*p* = 0.3385)	0.011	0.240
					CSA/control × *DAT*	F_2,204_ = 0.001 (*p* = 0.9996)	0.00001	0.050
BIS-MI	CSA; n = 100	21.58 ± 4.48	21.96 ± 4.27	21.67 ± 3.28	intercept	F_1,204_ = 3281.43 (*p* < 0.0001) *	0.942	1.000
	Control; n = 109	23.72 ± 4.29	24.30 ± 3.47	22.75 ± 5.23	CSA/control	F_1,204_ = 5.51 (*p* = 0.0199) *	0.027	0.647
					*DAT*	F_2,204_ = 0.50 (*p* = 0.6098)	0.005	0.131
					CSA/control × *DAT*	F_2,204_ = 0.16 (*p* = 0.8519)	0.002	0.075
BIS-NI	CSA; n = 100	25.20 ± 3.75	25.38 ± 4.03	25.56 ± 5.68	intercept	F_1,204_ = 5104.91 (*p* < 0.0001) *	0.962	1.000
	Control; n = 109	27.34 ± 3.83	27.70 ± 3.01	28.12 ± 6.47	CSA/control	F_1,204_ = 9.95 (*p* = 0.0018) *	0.047	0.881
					*DAT*	F_2,204_ = 0.21 (*p* = 0.8120)	0.002	0.082
					CSA/control × *DAT*	F_2,204_ = 0.03 (*p* = 0.9734)	0.003	0.054
BIS-Total	CSA; n = 100	62.87 ± 10.03	64.04 ± 9.63	63.55 ± 10.77	intercept	F_1,204_ = 5179.75 (*p* < 0.0001) *	0.962	1.000
	Control; n = 109	68.28 ± 10.17	70.30 ± 7.47	68.62 ± 13.81	CSA/control	F_1,204_ = 9.17 (*p* = 0.0028) *	0.043	0.854
					*DAT*	F_2,204_ = 0.63 (*p* = 0.5326)	0.006	0.155
					CSA/control × *DAT*	F_2,204_ = 0.05 (*p* = 0.9471)	0.0005	0.058
Number of methylation sites in the *DAT1* gene	CSA; n = 100	17.33 ± 5.59	18.93 ± 6.50	10.33 ± 5.45	intercept	F_1,204_ = 830.00 (*p* < 0.0001) *	0.803	1.000
	Control; n = 109	11.71 ± 3.28	11.87 ± 4.54	11.25 ± 4.03	CSA/control	F_1,204_ = 17.31 (*p <* 0.0001) *	0.078	0.985
					*DAT*	F_2,204_ = 6.05 (*p* = 0.0028) *	0.056	0.881
					CSA/control × *DAT*	F_2,204_ = 4.52 (*p* = 0.0120) *	0.042	0.766

*—significant result; CSA—combat sport athletes; M ± SD— mean ± standard deviation; n—number of subjects; *p*—statistical significance (ANOVA test); η^2^—effect size (partial eta squared).

**Table 5:** Post Hoc Analysis of the Interaction Between *DAT1* VNTR Genotype and Group on the Number of Methylation Sites in the *DAT1* Gene.

*DAT1* VNTR and Number of Methylation Sites in the *DAT1* Gene						
	{1} M = 17.33	{2} M = 18.93	{3} M = 10.33	{4} M = 11.71	{5} M = 11.87	{6} M = 11.25
CSA 10R/10R {1}		0.1258	0.0002 *	0.0001 *	0.0001 *	0.0017 *
CSA 9R/10R {2}			0.0001 *	0.0001 *	0.0001 *	0.0001 *
CSA 9R/9R {3}				0.44003	0.4031	0.7056
Control 10R/10R {4}					0.8703	0.8064
Control 9R/10R {5}						0.7466
Control 9R/9R {6}						

CSA—combat sport athletes; *—significant statistical differences, M—mean.

## Data Availability

The data presented in this study are available on request from the corresponding author due to privacy restrictions and in compliance with institutional and ethical regulations.

## Supplementary Materials

The following supporting information can be downloaded at https://www.mdpi.com/article/10.3390/biomedicines13122893/s1, Table S1: Methylation levels at individual CpG sites within the DAT1 gene in combat sport athletes and controls.
