# Supplementary material for: Interaction Between the VNTR of the DAT1 Gene and DAT1 Methylation in Relation to Impulsivity in Combat Sports Athletes
Source: Biomedicines. 2025 Nov 26;13(12):2893. doi: 10.3390/biomedicines13122893 (PMC12730208; doi:10.3390/biomedicines13122893)
Supplement: Supplementary file 1 [file biomedicines-13-02893-s001.zip › biomedicines-3859008-supplementary.pdf]

**Supplementary Table S1.** Methylation levels at individual CpG sites within the *DAT1* gene in combat sport athletes and controls.

| CpG site | Combat Sport Athletes and Control Participants | methylation level (%) | $\chi^2$ ($p$) | OR | 95% CI (-95%, +95%) |
|---|---|---|---|---|---|
| 1 | Combat sport athletes (100) | 73.0 | 45.61 (<0.001) | 7.55* | (4.09, 13.93) |
| | Control (109) | 26.4 | | | |
| 2 | Combat sport athletes (100) | 79.0 | 4.76 (0.029) | 1.98* | (1.07, 3.70) |
| | Control (109) | 65.5 | | | |
| 3 | Combat sport athletes (100) | 90.0 | 3.41 (0.065) | 0.34 | (0.10, 1.12) |
| | Control (109) | 96.4 | | | |
| 4 | Combat sport athletes (100) | 60.0 | 69.36 (<0.001) | 22.07* | (9.30, 52.36) |
| | Control (109) | 6.4 | | | |
| 5 | Combat sport athletes (100) | 55.0 | 17.90 (<0.001) | 3.41* | (1.91, 6.09) |
| | Control (109) | 26.4 | | | |
| 6 | Combat sport athletes (100) | 10.0 | 4.77 (0.029) | 1.68* | (1.21, 2.35) |
| | Control (109) | 2.7 | | | |
| 7 | Combat sport athletes (100) | 23.0 | 5.51 (0.019) | 2.44* | (1.14, 5.21) |
| | Control (109) | 10.9 | | | |
| 8 | Combat sport athletes (100) | 10.0 | 3.34 (0.067) | 2.92 | (0.88, 9.62) |
| | Control (109) | 3.7 | | | |
| 9 | Combat sport athletes (100) | 56.0 | 16.70 (<0.001) | 3.24* | (1.83, 5.75) |
| | Control (109) | 28.2 | | | |
| 10 | Combat sport athletes (100) | 58.0 | 13.53 (<0.001) | 2.84* | (1.62, 4.58) |
| | Control (109) | 32.7 | | | |
| 11 | Combat sport athletes (100) | 15.0 | 10.06 (0.001) | 6.29* | (1.76, 22.45) |
| | Control (109) | 2.7 | | | |
| 12 | Combat sport athletes (100) | 57.0 | 30.6 (<0.001) | 5.30* | (2.87, 9.78) |
| | Control (109) | 20.0 | | | |
| 13 | Combat sport athletes (100) | 20.0 | 16.02 (<0.001) | 8.92* | (2.56, 31.05) |
| | Control (109) | 2.7 | | | |
| 14 | Combat sport athletes (100) | 89.0 | 1.68 (0.198) | 1.69 | (0.76, 3.75) |
| | Control (109) | 82.7 | | | |
| 15 | Combat sport athletes (100) | 86.0 | 0.67 (0.411) | 1.36 | (0.65, 2.870 |
| | Control (109) | 81.8 | | | |
| 16 | Combat sport athletes (100) | 78.0 | 14.66 (<0.001) | 3.18* | (1.74, 5.81) |
| | Control (109) | 52.7 | | | |
| 17 | Combat sport athletes (100) | 44.0 | 10.74 (0.001) | 2.67* | (1.47, 4.85) |
| | Control (109) | 22.7 | | | |
| 18 | Combat sport athletes (100) | 16.0 | 9.29 (0.002) | 5.05* | (1.63, 15.66) |
| | Control (109) | 3.6 | | | |
| 19 | Combat sport athletes (100) | 99.0 | 1.57 (0.211) | 3.74 | (0.41, 34.00) |
| | Control (109) | 96.4 | | | |
| 20 | Combat sport athletes (100) | 55.0 | 26.07 (<0.001) | 4.62* | (2.52, 8.47) |
| | Control (109) | 20.9 | | | |
| 21 | Combat sport athletes (100) | 75.0 | 8.02 (0.005) | 2.32* | (1.29, 4.19) |
| | Control (109) | 56.4 | | | |

| | | | | | |
|---|---|---|---|---|---|
| 22 | Combat sport athletes (100) | 97.0 | 1.31 (0.253) | 2.20 | (0.55, 8.74) |
| | Control (109) | 93.6 | | | |
| 23 | Combat sport athletes (100) | 37.0 | 27.49 (<0.001) | 7.49* | (3.28, 17.11) |
| | Control (109) | 7.3 | | | |
| 24 | Combat sport athletes (100) | 76.0 | 3.27 (0.072) | 1.74 | (0.95, 3.18) |
| | Control (109) | 64.5 | | | |
| 25 | Combat sport athletes (100) | 63.0 | 40.20 (<0.001) | 6.81* | (3.67, 12.65) |
| | Control (109) | 20.0 | | | |
| 26 | Combat sport athletes (100) | 67.0 | 13.36 (<0.001) | 2.82* | (1.61, 4.96) |
| | Control (109) | 41.8 | | | |
| 27 | Combat sport athletes (100) | 42.0 | 32.58 (<0.001) | 8.13* | (3.69, 17.89) |
| | Control (109) | 8.2 | | | |
| 28 | Combat sport athletes (100) | 70 | 4.72 (0.030) | 1.87* | (1.06, 3.31) |
| | Control (109) | 55.5 | | | |
| 29 | Combat sport athletes (100) | 31.0 | 4.68 (0.031) | 2.02* | (1.06, 3.85) |
| | Control (109) | 18.2 | | | |
| 30 | Combat sport athletes (100) | 26.0 | 19.16 (<0.001) | 7.38* | (2.07, 20.10) |
| | Control (109) | 4.5 | | | |
| 31 | Combat sport athletes (100) | 9.0 | 3.83 (0.051) | 3.53 | (0.93, 13.42) |
| | Control (109) | 2.7 | | | |
| 32 | Combat sport athletes (100) | 70.0 | 0.49 (0.482) | 1.23 | (0.69, 2.20) |
| | Control (109) | 65.5 | | | |
| 33 | Combat sport athletes (100) | 80.0 | 0.62 (0.430) | 1.30 | (0.68, 2.50) |
| | Control (109) | 75.5 | | | |

*p* – statistical significance based on the $\chi^2$ test; OR – odds ratios, CI – 95% confidence intervals;
* – statistically significant differences.

**Supplementary Table 1 (ST1).**

Significantly higher methylation levels were observed at CpG sites 1, 2, 4–7, 9–13, 16–18, 20, 21, 23, and 25–30 in combat sport athletes compared with control participants ($p < 0.05$).

For the variables related to BIS-11 subscale scores and *DAT1* methylation site counts, the significance level was set at 0.01 (0.05/5) using the Bonferroni correction for multiple comparisons.

For the variables related to methylation of the 33 CpG sites presented in this table, a significance level of 0.0015 (0.05/33) was applied, also using the Bonferroni correction for multiple comparisons.
